# Prophylactic supplementation with *Bifidobacterium infantis* or its metabolite inosine attenuates cardiac ischemia/reperfusion injury

**DOI:** 10.1002/imt2.220

**Published:** 2024-07-02

**Authors:** Hao Zhang, Jiawan Wang, Jianghua Shen, Siqi Chen, Hailong Yuan, Xuan Zhang, Xu Liu, Ying Yu, Xinran Li, Zeyu Gao, Yaohui Wang, Jun Wang, Moshi Song

**Affiliations:** ^1^ Key Laboratory of Organ Regeneration and Reconstruction, State Key Laboratory of Membrane Biology Institute of Zoology, Chinese Academy of Sciences Beijing China; ^2^ University of Chinese Academy of Sciences Beijing China; ^3^ Department of Anesthesiology Beijing Chao‐Yang Hospital Beijing China; ^4^ Beijing Institute for Stem Cell and Regenerative Medicine Beijing China; ^5^ Joint National Laboratory for Antibody Drug Engineering Henan University Kaifeng China; ^6^ CAS Key Laboratory of Pathogenic Microbiology and Immunology, Institute of Microbiology Chinese Academy of Sciences Beijing China; ^7^ Institute for Stem Cell and Regeneration, Chinese Academy of Sciences Beijing China

**Keywords:** *Bifidobacterium infantis*, cardioprotection, inosine, myocardial ischemia–reperfusion

## Abstract

Emerging evidence has demonstrated the profound impact of the gut microbiome on cardiovascular diseases through the production of diverse metabolites. Using an animal model of myocardial ischemia–reperfusion (I/R) injury, we found that the prophylactic administration of a well‐known probiotic, *Bifidobacterium infantis* (*B. infantis*), exhibited cardioprotective effects in terms of preserving cardiac contractile function and preventing adverse cardiac remodeling following I/R and that these cardioprotective effects were recapitulated by its metabolite inosine. Transcriptomic analysis further revealed that inosine mitigated I/R‐induced cardiac inflammation and cell death. Mechanistic investigations elucidated that inosine suppressed the production of pro‐inflammatory cytokines and reduced the numbers of dendritic cells and natural killer cells, achieved through the activation of the adenosine A2A receptor (A2AR) that when inhibited abrogated the cardioprotective effects of inosine. Additionally, in vitro studies using C2C12 myoblasts revealed that inosine attenuated cell death by serving as an alternative carbon source for adenosine triphosphate (ATP) generation through the purine salvage pathway when subjected to oxygen‐glucose deprivation/reoxygenation that simulated myocardial I/R injury. Likewise, inosine reversed the I/R‐induced decrease in ATP levels in mouse hearts. Taken together, our findings indicate that *B. infantis* or its metabolite inosine exerts cardioprotective effects against I/R by suppressing cardiac inflammation and attenuating cardiac cell death, suggesting prophylactic therapeutic options for acute ischemic cardiac injury.

## Highlights

- *Bifidobacterium infantis*, a well‐known probiotic, exhibits prophylactic cardioprotective effects against myocardial I/R injury.
- The cardioprotective effects of *Bifidobacterium infantis* are recapitulated by its metabolite inosine.
- Inosine treatment suppresses cardiac inflammation by reducing the production of pro‐inflammatory cytokines and decreasing the numbers of dendritic cells and natural killer cells after I/R.
- Inosine treatment attenuates cell death by serving as an alternative carbon source for adenosine triphosphate generation through the purine salvage pathway in stressed myocytes and in I/R‐injured mouse hearts.

## Graphical abstract

In this study, we found that the prophylactic administration of a well‐known probiotic, *Bifidobacterium infantis* (*B. infantis*), exhibited cardioprotective effects against myocardial ischemia–reperfusion (I/R) injury, which was recapitulated by its metabolite inosine. Specifically, inosine suppressed cardiac inflammation by reducing the production of pro‐inflammatory cytokines and decreasing the numbers of dendritic cells and natural killer cells after I/R. Additionally, inosine attenuated cell death by serving as an alternative carbon source for adenosine triphosphate generation through the purine salvage pathway in stressed myocytes and in I/R‐injured mouse hearts. Collectively, our findings indicate that *B. infantis* or its metabolite inosine exerts pleiotropic cardioprotective effects against I/R, suggesting prophylactic therapeutic options for acute ischemic cardiac injury.

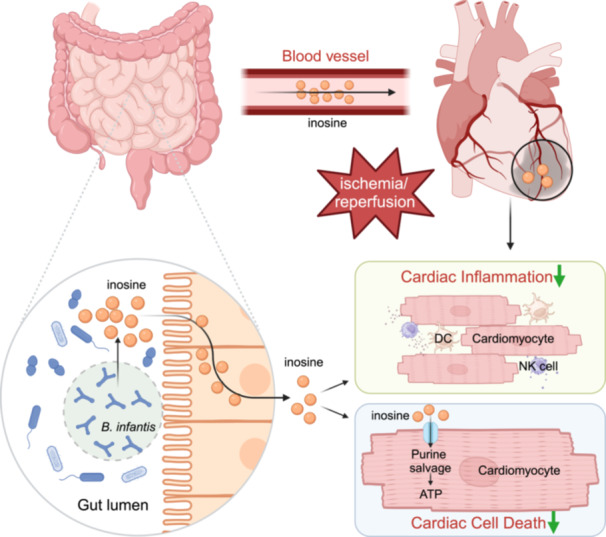

## INTRODUCTION

Despite the significant advances in therapeutic strategies, cardiovascular disease (CVD), encompassing ischemic heart disease and stroke, continues to be a leading global cause of morbidity and mortality [1]. The chronic progression of CVD necessitates the search for prophylactic and therapeutic interventions for patients suffering from these conditions. Among CVD manifestations, myocardial infarction (MI) represents the most severe form, often accompanied by the severe complications of ischemia/reperfusion (I/R) injury. This injury, often a consequence of treatments for MI, accounts for approximately half of all final infarct cases [2, 3]. I/R injury typically occurs after percutaneous coronary intervention and thrombolytic therapy and involves a complex interplay of metabolic disturbances, inflammation, oxidative stress, and microvascular obstruction [4]. The global challenge posed by MI and I/R injury highlights the urgency for novel and more effective therapeutic strategies.

A growing number of studies underscores the pivotal role of the gut‐heart axis, where the gut microbiome and its metabolites are intricately linked to a spectrum of CVDs, ranging from hypertension [5, 6] to heart failure [7, 8], and, recently, MI [9, 10]. Besides the production of metabolites such as trimethylamine (TMA) [6, 11], short‐chain fatty acids (SCFAs) [12, 13], bile acids [14, 15], and succinates [16, 17], the gut microbiome also plays a crucial role in regulating the host′s immune and inflammatory responses via the modulation of immune cells, including regulatory T cells, innate lymphatic cells, and macrophages. The gut microbiome is already known to shape the immune landscape and affect disease onset and progression of metabolic and immune diseases [18, 19, 20], and in stroke, the gut microbiome has been shown to modulate patient outcomes also through immune cells [21, 22]. In recognition of the role of the gut microbiome in regulating host metabolic and immune functions, probiotics have garnered significant attention for their potential in preventing or treating various diseases [23]. Commonly used in the fermentation of dairy products, several strains of probiotics, including but not limited to *Bifidobacterium animalis, Lactobacillus gasseri*, and *Lactiplantibacillus plantarum*, have been evaluated for their efficacy in treating obesity, nonalcoholic liver diseases (NASH), and type 2 diabetes [24, 25]. Additionally, novel probiotic species such as *Akkermansia muciniphila* have emerged, demonstrating promising results in human clinical trials targeting metabolic disorders [26]. However, little is known about the protective effects of probiotics in the case of MI and I/R.

*Bifidobacteria*, a component of the intestinal microbiota in mammals and other animals, have exhibited remarkable efficacy in alleviating symptoms such as constipation, abdominal pain, flatulence, and bloating. This therapeutic effect is attributed to their ability to restore gut flora balance and inhibit abnormal bacterial fermentation of food residues [27]. Notably, several studies have demonstrated a significant decrease in the abundance of *Bifidobacterium* genus among MI patients [28]. *Bifidobacterium infantis* (*B. infantis*), as a commonly used probiotic species, has been reported to protect against pathogenic infections and mitigate inflammation in a range of gastrointestinal disorders such as ulcerative colitis and non‐gastrointestinal disorders, including chronic fatigue syndrome and psoriasis [29], highlighting its potential to resolve inflammation in diseased conditions. Specifically, patients with coronary artery disease (CAD) exhibit a lower abundance of *B. infantis* compared to healthy individuals [30]. However, it remains unclear whether *B. infantis* may affect the prognosis of CAD, particularly in the context of myocardial I/R injury.

Here, we investigated the prophylactic effects of *B. infantis* supplementation in a mouse model of myocardial I/R injury. Our study demonstrated that *B. infantis* had protective effects on heart injury against I/R, in particular through its metabolite inosine, which has a combinatory effect of reducing cardiac inflammation while attenuating cardiac cell death, thereby demonstrating the potential of *B. infantis* or its metabolite inosine as promising therapeutic agents in the management of myocardial I/R injury.

## RESULTS

### The gavage of *B. infantis* attenuated the cardiac injury after I/R

**Figure 1:**
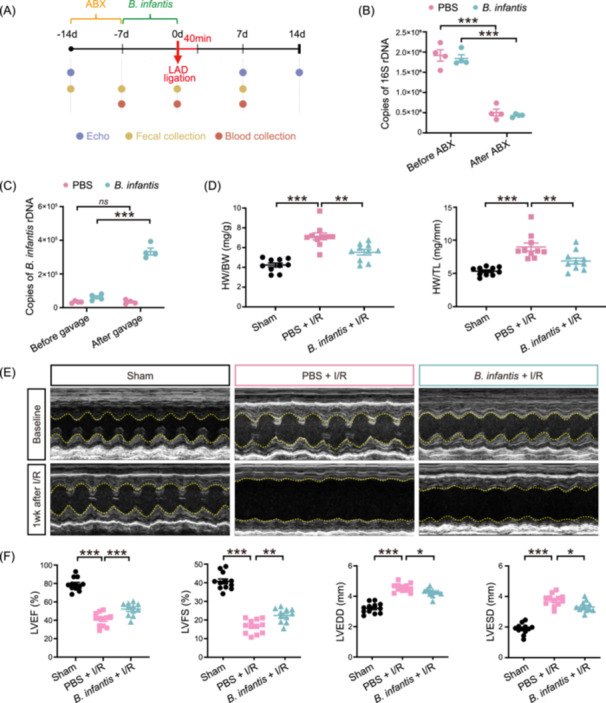
The gavage of *Bifidobacterium infantis* mitigated cardiac injury in mouse hearts following ischemia/reperfusion (I/R). (A) Schematic diagram of the experiment with *B. infantis* gavage. Male C57BL/6J mice were pretreated with 7 days of antibiotics to remove gut microbiota and 7 days of *B. infantis* gavage before I/R. Serum samples were collected 7 days before I/R, 2 days, and 7 days after I/R. Fecal samples were collected 7 days before I/R and 7 days after I/R. Cardiac function was detected by transthoracic echocardiography (Echo) at baseline, 7 days before I/R, 3 days before I/R, 7 days after I/R, and 14 days after I/R. (B) The copies of bacterial 16S rDNA were detected in the feces collected before and after antibiotics (ABX) treatment. Data are shown as the mean ± SEMs, *n* = 4 per group. ***p < 0.001 (two‐way ANOVA). (C) The copies of *B. infantis* rDNA were detected in the feces collected before and after 7 days of *B. infantis* gavage treatment. Data are shown as the mean ± SEMs, *n* = 4 per group. *ns*, not significant; ***p < 0.001 (two‐way ANOVA). (D) The ratio of heart weight to body weight (HW/BW) and heart weight to tibial length (HW/TL). Data are shown as the mean ± SEMs. *n* = 10 per group. **p < 0.01; ***p < 0.001 (one‐way ANOVA with post hoc Tukey test). (E) Representative echocardiographic images at baseline and 7 days after I/R. (F) Quantitative data of left ventricular ejection fraction (LVEF), left ventricular fractional shortening (LVFS), left ventricular end‐diastolic dimension (LVEDD), and left ventricular end‐systolic dimension (LVESD) 7 days after I/R are shown as the mean ± SEMs. Sham group (Sham), *n* = 12; PBS‐gavaged I/R group (PBS + I/R), *n* = 12; *B. infantis*‐gavaged I/R group (*B. infantis* + I/R), *n* = 11. *p < 0.05; **p < 0.01; ***p < 0.001 (one‐way ANOVA with post hoc Dunnett′s test). LAD, left anterior descending.

Previous studies described the anti‐inflammatory and metabolic‐modulatory effects of *B. infantis* [29, 31]. In this study, the possible role of *B. infantis* gavage in heart protection from acute ischemic cardiac injury was investigated (Figure 1A). Following the depletion of the gut microbiota using orally administered antibiotics for 7 days (Figure 1B), 8‐week‐old male C57BL/6J mice were subjected to a daily gavage of *B. infantis* (2 × 10^7^ CFU/day) or PBS (control group) for an additional 7 days. The colonization of the *B. infantis* was confirmed by 16S rDNA quantification (*p* < 0.001) (Figure 1C), followed by the induction of myocardial I/R through the temporary ligation of the left anterior descending coronary artery for a total of 40 min and the timely opening of the occluded artery for reperfusion of the ischemic tissue, using a sham operation group as the control group. After 7‐day post‐surgery, a significant hypertrophic response in PBS‐gavaged I/R mice was observed when compared to the sham group, as reflected by a 50% increase in heart‐to‐body weight ratio (*p* < 0.001) and heart‐to‐tibia length ratio (*p* < 0.001), which was attenuated in *B. infantis*‐gavaged I/R mice (*p* < 0.01 and *p* < 0.01, respectively) (Figure 1D).

**Figure 2:**
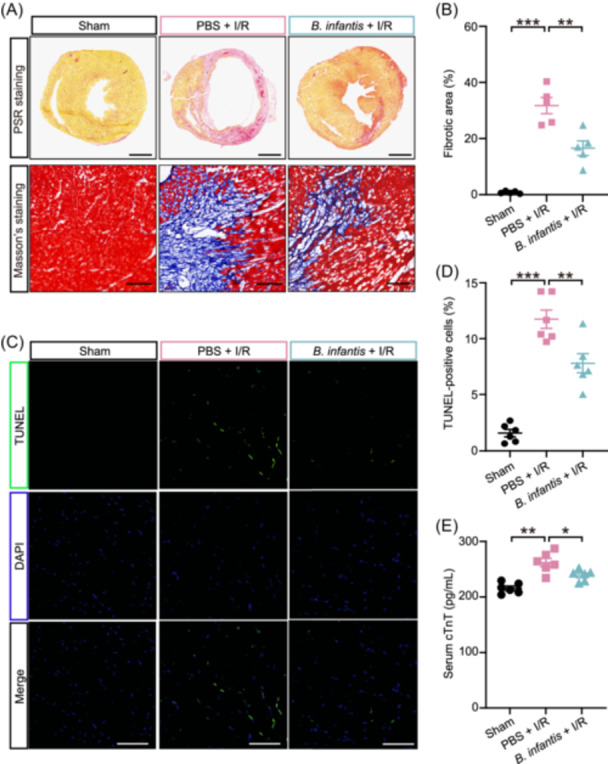
The gavage of *Bifidobacterium infantis* decreased cardiac fibrosis and cell apoptosis in mouse hearts following I/R. (A) Picrosirius red (PSR) staining of the midventricular section (the top panel) and Masson's trichrome staining (the bottom panel) of heart tissues at the border zones of the infarcted area 7 days after I/R surgery. For PSR staining, the red areas represent collagen. Scale bar, 1 mm. For Masson's staining, the blue signals indicate the fibrotic areas. Scale bar, 100 μm. (B) Quantification of the fibrotic area. Data are shown as the mean ± SEMs; *n* = 5 per group. **p < 0.01; ***p < 0.001 (one‐way ANOVA with post hoc Tukey test). (C) Evaluation of apoptotic cardiomyocytes in mouse hearts co‐stained with TUNEL (green). Scale bar, 50 μm. (D) The average number of apoptotic cells per field at 200x magnification. n = 6 per group; **p < 0.01; ***p < 0.001 (one‐way ANOVA with post hoc Tukey test). (E) Mouse serum cTnT measured by ELISA 3 days after I/R. *n* = 6 per group. *p < 0.05; **p < 0.01 (one‐way ANOVA with post hoc Tukey test). DAPI, 4′,6‐diamidino‐2‐phenylindole; I/R, ischemia/reperfusion; TUNEL, Terminal deoxynucleotidyl transferase dUTP nick end labeling.

In addition, *B. infantis*‐gavaged I/R mice were shown to have well‐preserved cardiac functions when compared to PBS‐gavaged I/R mice by echocardiography at 1 week (Figure 1E,F) and 2 weeks (Supporting Information S1: Figure S1A,B) post‐surgery. Compared to PBS‐gavaged I/R group, *B. infantis*‐gavaged I/R group exhibited significantly higher percentages of left ventricular ejection fraction (LVEF%; *p* < 0.001 for 1 week and *p* < 0.001 for 2 weeks), fractional shortening (LVFS%; *p* < 0.01 for 1 week and *p* < 0.01 for 2 weeks), and significantly lower left ventricular end‐diastolic dimension (LVEDD; *p* < 0.05 for 1 week and *p* < 0.001 for 2 weeks) and left ventricular end‐systolic dimension (LVESD; *p* < 0.05 for 1 week and *p* < 0.001 for 2 weeks) (Figure 1E,F). These results demonstrate that the gavage of *B. infantis* plays a role in the prevention of left ventricular dilatation and the preservation of cardiac function following I/R.

### The gavage of *B. infantis* reduced cardiac cell death and fibrosis after I/R

The characterization of the histologic characteristics of myocardial I/R is crucial to understanding the pathophysiology underlying the morphological changes from the initial infarction and reperfusion to chronic fibrosis [32, 33]. We next performed histopathologic examination of the tissues collected from *B. infantis*‐gavaged mice and PBS‐gavaged after I/R. Bigger scar sizes and more fibrotic tissues were detected by Picrosirius red staining and Masson's trichrome staining in the heart samples collected from PBS‐gavaged I/R mice (*p* < 0.001); however, they both were significantly reduced in the hearts of *B. infantis*‐gavaged I/R group (*p* < 0.01) (Figure 2A,B). It was also found that the gavage of *B. infantis* resulted in less I/R‐induced cardiac cell death, as evidenced by an increased TUNEL positivity in PBS‐gavaged I/R mice when compared to the sham group (*p* < 0.001) as well as by the reversed TUNEL positivity in *B. infantis*‐gavaged I/R mice (*p* < 0.01) (Figure 2C,D). Consistently, the serum cardiac troponin T (cTnT), which is an established biomarker of cardiomyocyte damage, significantly increased after I/R (*p* < 0.01) but it decreased after the gavage of *B. infantis* (*p* < 0.05) (Figure 2E). Therefore, our results further prove that *B. infantis* exerts a cardioprotective effect through the reduction of I/R‐induced cardiac fibrosis and cell death.

### Inosine recapitulated the cardioprotective effect of *B. infantis* after I/R

**Figure 3:**
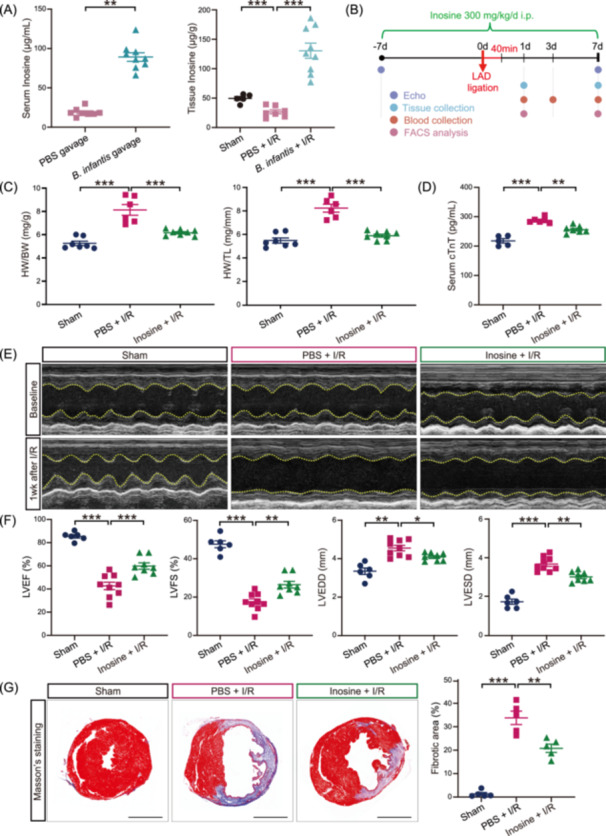
Inosine recapitulated the cardioprotective effects of *Bifidobacterium infantis* in mouse hearts against I/R. (A) Levels of inosine were detected in the serum (left panel) 7 days after continuous *B. infantis* gavage, and levels of inosine in the heart tissues (right panel) 14 days after I/R by HPLC‐MS. Data are shown as the mean ± SEMs. PBS‐gavaged group (PBS), *n* = 8; *B. infantis*‐gavaged group (*B. infantis*), *n* = 9 in the left panel. **p < 0.01 (Student's t test). Sham group (Sham), *n* = 5; PBS‐gavaged group (PBS), *n* = 7, *B. infantis*‐gavaged group (*B. infantis*), *n* = 9 in the right panel. ***p < 0.001 (Student's t test). (B) Schematic diagram of the mouse experiment with inosine treatment. Male C57BL/6J mice were treated with inosine via intraperitoneal injection for 7 days before I/R and then continued injection once per day for another 7 days after I/R. Cardiac function was detected by echocardiogram before and 7 days after I/R. Serum samples were collected 1 and 7 days after I/R. Cardiac tissues were collected 1 and 7 days after I/R. (C) The ratios of heart weight to body weight (HW/BW) and heart weight to tibial length (HW/TL). Data are shown as the mean ± SEMs. Sham group (Sham), *n* = 7; PBS‐treated group (PBS + I/R), *n* = 6; Inosine‐treated group (Inosine + I/R), *n* = 8. ***p < 0.001 (one‐way ANOVA with post hoc Dunnett's test). (D) Mouse serum cTnT measured by ELISA 3 days after I/R. Sham group (Sham), *n* = 5; PBS‐treated I/R group (PBS + I/R), *n* = 6; inosine‐treated I/R group (Inosine + I/R), *n* = 7. **p < 0.01; ***p < 0.001 (one‐way ANOVA with post hoc Dunnett′s test). (E) Representative echocardiographic images at baseline and 7 days after I/R. (F) Quantitative results of LVEF, LVFS, LVEDD, and LVESD are shown as the mean ± SEMs. Sham group (Sham), *n* = 6; PBS‐treated I/R group (PBS + I/R), *n* = 9; inosine‐treated I/R group (Inosine + I/R), *n* = 8. *p < 0.05; **p < 0.01; ***p < 0.001 (one‐way ANOVA with post hoc Dunnett′s test). (G) Masson's trichrome staining of mouse hearts 7 days after I/R surgery. Blue signals indicate the fibrotic areas. Scale bar, 2 mm. Quantification data of the fibrotic area are shown to the right as the mean ± SEMs; *n* = 5 per group. **p < 0.01; ***p < 0.001 (one‐way ANOVA with post hoc Tukey test). i.p., intraperitoneal; I/R, ischemia/reperfusion; LVEF, left ventricular ejection fraction; LVFS, left ventricular fractional shortening; LVEDD, left ventricular end‐diastolic dimension; LVESD, left ventricular end‐systolic dimension.

In light of the fact that previous studies reported extensive inosine production and secretion by *B. infantis* [34, 35, 36], we postulated that inosine may play a role in the cardioprotective effect of the gavage of *B. infantis.* We first evaluated whether the antibiotic treatment may affect serum inosine levels in mice and found no significant differences before or after the antibiotic treatment (Supporting Information S1: Figure S2). Next, we measured the inosine levels in PBS‐gavaged I/R and *B. infantis*‐gavaged I/R groups, and we found a significant increase of inosine levels in both serum and heart tissues of *B. infantis*‐gavaged mice when compared to those found in PBS‐gavaged mice (*p* < 0.01 and *p* < 0.001, respectively) (Figure 3A).

Next, we investigated the hypothesized cardioprotective effects through intraperitoneal injection of inosine in I/R mice (Figure 3B). We found that inosine treatment recapitulated the beneficial effects of the *B. infantis* gavage mice. Specifically, inosine significantly suppressed the I/R‐induced cardiac hypertrophy, as evidenced by the increased heart‐to‐body weight ratio and heart‐to‐tibia length ratio following I/R (*p* < 0.001 and *p* < 0.001, respectively) that decreased by inosine treatment (*p* < 0.001 and *p* < 0.001, respectively) (Figure 3C). Consistently, we observed that the concentration of serum cTnT significantly increased after I/R (*p* < 0.001) but decreased by inosine treatment (*p* < 0.01) (Figure 3D). In addition, inosine mitigated cardiac dysfunction induced by I/R, as evidenced by significantly higher percentages of LVEF (*p* < 0.001), LVFS (*p* < 0.01), and significantly lower LVEDD (*p* < 0.05) and LVESD (*p* < 0.01) (Figure 3E,F). More fibrotic tissues were detected by Masson's trichrome staining in the heart samples collected from I/R mice (*p* < 0.001); however, they were significantly reduced in the hearts of inosine‐treated I/R group (*p* < 0.01) (Figure 3G). Therefore, our data suggest that inosine recapitulates the cardioprotective effects of *B. infantis* after I/R.

### Transcriptional analysis revealed improved energy metabolism and suppressed immune response and cell apoptosis by inosine in hearts following I/R

**Figure 4:**
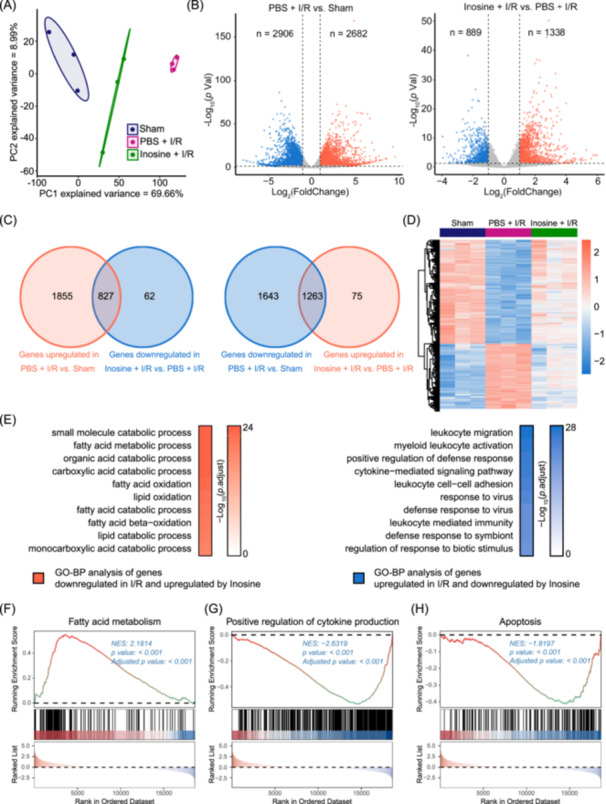
Transcriptional analysis revealing the improved energy metabolism and suppressed immune response in mouse hearts subjected to I/R surgery and inosine treatment. (A) Principal component analysis (PCA) revealing in‐group clusters with minimal overlap. (B) Volcano plot illustrating differentially expressed genes identified between Sham and PBS + I/R group (left panel) and between Inosine + I/R and PBS + I/R group (right panel). The blue dots denote downregulated gene expression, the red dots denote upregulated gene expression, and the gray dots denote gene expression without statistical difference. |Fold change| > 2, adjusted *p* value < 0.05. (C) Left, Venn diagram showing the overlap between significantly upregulated genes in PBS + I/R versus Sham and significantly downregulated genes in Inosine + I/R versus PBS + I/R. Right, Venn diagram showing the overlap between significantly downregulated genes in PBS + I/R versus Sham and significantly upregulated genes in Inosine + I/R versus PBS + I/R. Differentially expressed genes were identified with a cutoff of |Fold change| > 2 and adjusted *p* value < 0.05. (D) Heatmap of the expression levels of genes differentially expressed in PBS + I/R versus Sham and rescued in Inosine + I/R versus PBS + I/R. The expression level for each gene is represented by a color range from blue (low) to red (high). (E) Left, top 10 enriched gene ontology biological processes (GO‐BP) terms of genes downregulated in I/R versus Sham and upregulated in Inosine + I/R versus PBS + I/R (red). Right, the top 10 enriched GO‐BP terms of genes upregulated in I/R versus Sham and downregulated in Inosine + I/R versus PBS + I/R (blue) are listed. (F–H) Gene set enrichment analysis (GSEA) plots showing the enrichment of gene sets related to fatty acid metabolism (F), positive regulation of cytokine production (G), and apoptosis (H) in the Inosine + I/R when compared to the PBS + I/R group. I/R, ischemia/reperfusion.

To better understand the mechanism underlying the protection of inosine treatment against cardiac I/R injury, we performed a transcriptional analysis of infarcted heart tissues of the inosine‐treated I/R group and PBS‐treated I/R group 1 day after I/R, along with the heart tissues of the sham group by RNA sequencing (Figure 4A). A total of 2682 upregulated and 2906 downregulated genes were found in the PBS‐treated I/R group (Figure 4B), out of which 827 I/R‐upregulated and 1263 I/R‐downregulated genes were rescued by inosine treatment (Figure 4C,D). Subsequently, a gene ontology (GO) enrichment analysis identified the biological processes mostly upregulated and downregulated by inosine treatment (Figure 4E). Out of the top 10 significantly upregulated pathways, 8 were associated with fatty acid catabolism, suggesting that inosine positively reverses the IR‐induced adverse metabolic reprogramming from fatty acid metabolism to glycolysis. Further, a gene set enrichment analysis (GSEA) revealed that inosine improved the impaired cardiac energy metabolism after I/R, as shown by fatty acid metabolism, which was downregulated after I/R but rescued by inosine treatment (Figure 4F and Supporting Information S1: Figure S3A), as shown by the genes related to this process, such as *Ces1d*, *Acad11*, *Cpt2*, *Ehhadh*, and *Acadm* (Supporting Information S1: Figure S3B). Conversely, out of the 10 pathways mostly downregulated by inosine treatment, 9 were associated with inflammatory response pathways such as cytokine‐mediated signaling pathway and leukocyte cell–cell adhesion, highly suggestive of an anti‐inflammatory cardioprotective effect of inosine (Figure 4E). Similarly, GSEA data also showed that the positive regulation of cytokine production was upregulated after I/R and rescued by inosine treatment (Figure 4G and Supporting Information S1: Figure S3A), as shown by genes related to this process, including *Trem1, Il‐1b*, *Il‐6*, *Inava* and *Il18r1* (Supporting Information S1: Figure S3C). Furthermore, GSEA data revealed that apoptosis was upregulated after I/R and rescued by inosine treatment (Figure 4H and Supporting Information S1: Figure S3A), as shown by the genes related to this process, such as *Bcl2a1a*, *Pmaip1*, *Traf1*, *Bid*, and *Tnfrsf10b* (Supporting Information S1: Figure S3D)*.* Therefore, our transcriptional data suggest that inosine confers cardioprotection by the improvement of energy metabolism as well as the attenuation of cardiac inflammation and cell apoptosis.

### Inosine attenuated the cardiac inflammation by inhibiting the pro‐inflammatory response in mouse hearts after I/R via adenosine A2A receptor (A2AR) activation

**Figure 5:**
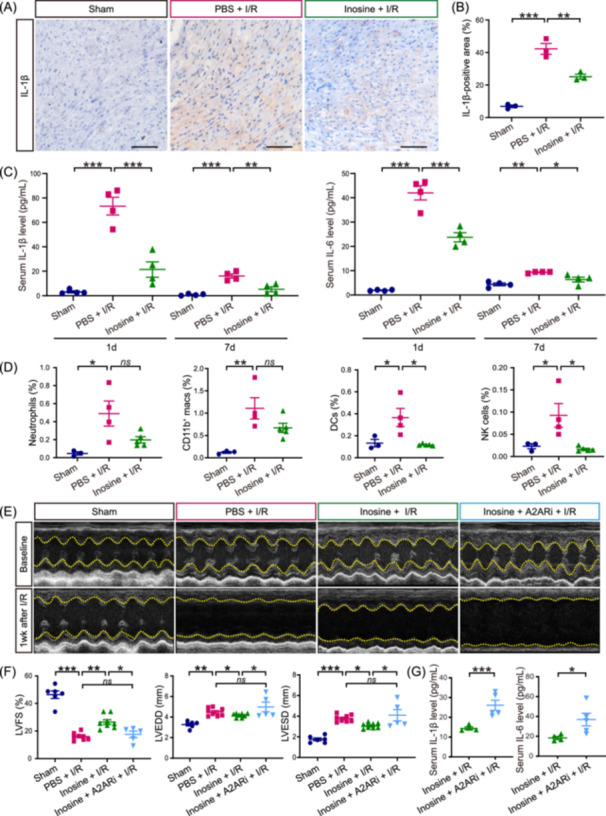
Inosine reduced cardiac inflammation after I/R via the activation of the anti‐inflammatory adenosine A2A receptor. (A) Representative images of immunohistochemistry staining for IL‐1β in mouse heart tissues after I/R. The brown signal indicates the cells positively express IL‐1β. Scale bar, 50 μm. (B) Quantitative data of IL‐1β in mouse heart tissues after I/R in (A). Data are shown as the mean ± SEMs, *n* = 3 per group. **p < 0.01; ***p < 0.001 (one‐way ANOVA with post hoc Tukey test). (C) Serum IL‐1β (left panel) and IL‐6 (right panel) levels measured by ELISA 1 and 7 days after I/R. *n* = 4 per group. *p < 0.05, **p < 0.01; ***p < 0.001 (one‐way ANOVA with post hoc Tukey test). (D) Quantitative results of neutrophils, CD11b^+^ macrophages (macs), dendritic cells (DCs), and natural killer (NK) cells in three groups 7 days after I/R are shown as the mean ± SEMs. Sham group (Sham), *n* = 3; PBS‐treated I/R group (PBS + I/R), *n* = 4; inosine‐treated I/R group (Inosine + I/R), *n* = 5. *ns*, not significant; *p < 0.05; **p < 0.01 (one‐way ANOVA with post hoc Dunnett's test). (E) Representative echocardiographic images at baseline and 7 days after I/R. (F) Quantitative results of LVFS, LVEDD, and LVESD are shown as the mean ± SEMs to the right. Sham group (Sham), *n* = 6; PBS‐treated I/R group (PBS + I/R), *n* = 8; inosine‐treated I/R group (Inosine + I/R), *n* = 8; inosine‐ and A2ARi‐treated group (Inosine + A2ARi + I/R), *n* = 5. *ns*, not significant; *p < 0.05; **p < 0.01; ***p < 0.001 (one‐way ANOVA with post hoc Dunnett's test). (G) Serum inflammatory cytokines IL‐1β and IL‐6 measured by ELISA 7 days after I/R. Inosine‐treated I/R group (Inosine + I/R), *n* = 4; inosine‐ and A2ARi‐treated group (Inosine + A2ARi + I/R), *n* = 5. *p < 0.05; ***p < 0.001 (Student's t test). A2ARi, A2AR inhibitor (ZM241385); I/R, ischemia/reperfusion; LVFS, left ventricular fractional shortening; LVEDD, left ventricular end‐diastolic dimension; LVESD, left ventricular end‐systolic dimension.

Cardiac inflammation, exclusively regulated by a variety of cytokines and immune cells, plays a crucial role in the regulation of pathophysiological processes and in the prognosis of ischemic‐reperfused hearts [37, 38, 39]. Therefore, the modulation of the inflammatory response became a promising target for the treatment of cardiac injury and enhancement of cardiac function following MI. Since the RNA sequencing data from this study demonstrated an anti‐inflammation effect by inosine treatment after I/R, we examined the expression of interleukin‐1β (IL‐1β), which is an early and chronically I/R‐upregulated cytokine [40]. We found that the I/R‐induced elevated *Tnf* levels by qPCR and increased IL‐1β expression by immunohistochemistry (*p* < 0.001 and *p* < 0.001, respectively) were both attenuated by inosine treatment (*p* < 0.01 and *p* < 0.01, respectively) (Figure 5A,B and Supporting Information S1: Figure S4). Similarly, we found by ELISA that the concentrations of circulating pro‐inflammatory cytokines, IL‐1β and IL‐6, increased after I/R (*p* < 0.001, *p* < 0.001, *p* < 0.001, and *p* < 0.01, respectively) and were suppressed by inosine treatment (*p* < 0.001, *p* < 0.01, *p* < 0.001 and *p* < 0.05, respectively) 1 and 7 days after I/R (Figure 5C), suggesting that inosine reduces the levels of cardiac and systemic inflammation following I/R.

To better understand how inosine treatment mitigated the I/R‐induced inflammation, we performed a flow cytometry analysis of the immune cells in mouse hearts 1 day (Supporting Information S1: Figure S5A,B) and 7 days after I/R (Figure 5D and Supporting Information S1: Figure S5C). We found that the proportion of neutrophils (CD45^+^CD11b^+^Ly6G^high^) and CD11b^+^ macrophages (CD45^+^CD11b^+^F4/80^+^) significantly increased 1 day after I/R (*p* < 0.05 and *p* < 0.05, respectively), while inosine did not affect the proportion of neither neutrophils nor CD11b^+^ macrophages. However, they decreased in the inosine‐treated I/R group 7 days after I/R compared with the PBS + I/R group (*p* = 0.0711 and *p* = 0.1542, respectively). In addition, dendritic cells (DCs) (gated by CD45^+^CD11c^+^I‐Ab^+^Ly6C^−^Ly6G^−^) and natural killer (NK) cells (gated by CD45^+^NK1.1^+^CD3^−^) were significantly increased (*p* < 0.05 and *p* < 0.05, respectively), but significantly reduced by inosine treatment (*p* < 0.05 and *p* < 0.05, respectively) (Figure 5D and Supporting Information S1: Figure S5C), highlighting the role of inosine as an anti‐inflammatory agent for multiple types of immune cells after cardiac I/R.

Previous studies have reported that inosine exerts broad‐spectrum anti‐inflammatory and immunomodulatory effects through adenosine receptors (ARs) [41, 42]. Given that the adenosine A2A receptor is the predominant type of AR receptor expressed on several immune cell types, including neutrophils, monocytes/macrophages, DCs, as well as NK and NKT cells [43], we postulated that inosine might exert its effects through A2AR. To test this hypothesis, we administered a pharmacological inhibitor of the inosine‐A2AR pathway, ZM241385 (A2ARi), before the I/R surgery. Our results revealed that in the presence of A2ARi, inosine failed to confer cardioprotection in I/R hearts (Figure 5E,F). Additionally, analysis of circulating pro‐inflammatory cytokines such as IL‐1β and IL‐6 using ELISA revealed that inosine treatment did not suppress their levels (Figure 5G), demonstrating that the inosine's effects in reducing the cardiac injury and inflammation after I/R are dependent on A2AR activation.

### Inosine attenuated cardiac cell death by serving as an alternative source for ATP generation through the purine salvage pathway

**Figure 6:**
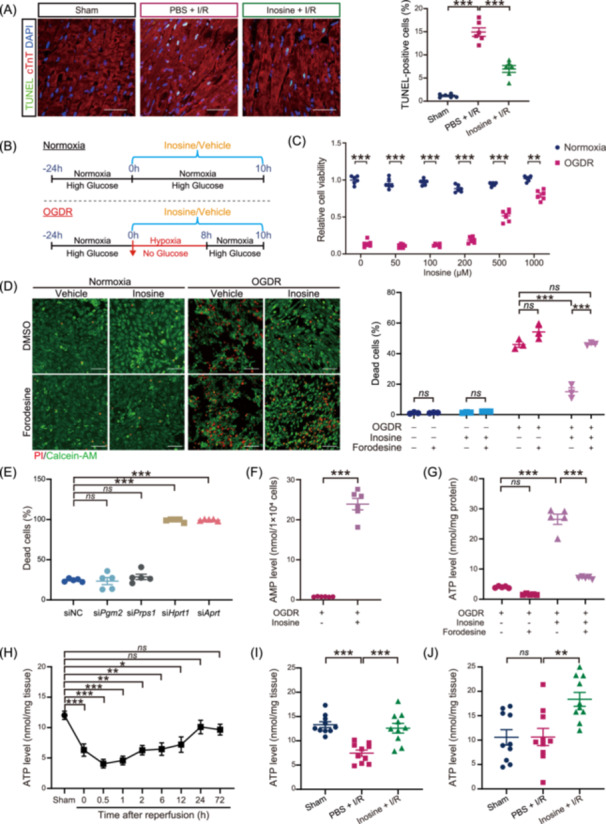
Inosine attenuated cardiac cell death by serving as an alternative carbon source for ATP generation through the purine salvage pathway. (A) Evaluation of apoptotic cardiomyocytes at the border zone of mouse hearts subjected to I/R surgery and co‐stained with TUNEL (green) and cardiac troponin T (red). Scale bar, 50 μm. Quantitative data are shown as the mean ± SEMs. *n* = 6 per group. ***p < 0.001 (one‐way ANOVA with post hoc Tukey test). (B) Schematic diagram of oxygen‐glucose deprivation/reoxygenation (OGDR) induction and inosine treatment in C2C12 myoblasts. (C) Cell viability detected by Alamar Blue of C2C12 myoblasts treated with different concentrations of inosine under normoxic and OGDR conditions. *n* = 6 per group. **p < 0.01; ***p < 0.001 (Student's t test). (D) Measurement of cell death by PI/Calcein‐AM staining in C2C12 myoblasts treated with inosine and DMSO or forodesine under normoxic and OGDR conditions. The red dots denote dead cells, and the green dots denote live cells. Scale bar, 100 μm. *n* = 3 per group. ***p < 0.001 (two‐way ANOVA). (E) Quantitative data of cell death measured by PI/Calcein‐AM staining in C2C12 myoblasts with siRNA‐mediated knockdown of genes related to purine salvage pathway in the presence of inosine under OGDR condition. *n* = 5 per group. ***p < 0.001 (one‐way ANOVA with post hoc Tukey test). (F) Levels of AMP measured in C2C12 myoblasts in the presence or absence of inosine under OGDR condition. *n* = 6 per group. ***p < 0.001 (Student's t test). (G) Levels of ATP measured in C2C12 myoblasts treated with DMSO or forodesine in the presence or absence of inosine under OGDR condition. *n* = 5 per group. ***p < 0.001 (one‐way ANOVA with post hoc Tukey test). (H) Levels of ATP measured in heart tissues of the inosine‐treated group at different times after IR. *n* = 6 per group. Data are shown as the mean ± SEMs. *p < 0.05; **p < 0.01; ***p < 0.001 (Student's t test). (I, J) Levels of ATP measured in heart tissues at 30 min (I) and 24 h (J) after I/R. *n* = 10 per group. Data are shown as the mean ± SEMs. **p < 0.01; ***p < 0.001 (one‐way ANOVA with post hoc Tukey test). AMP, adenosine monophosphate; ATP, adenosine triphosphate; DMSO, dimethylsulfoxide; I/R, ischemia/reperfusion; *ns*, not significant; PI, propidium iodide; TUNEL, terminal deoxynucleotidyl transferase dUTP nick end labeling.

Besides the modulation of cardiac inflammation, the RNA sequencing data from this study also suggested that inosine treatment attenuated cardiac injury by inhibiting the I/R‐induced cardiac cell apoptosis. Accordingly, we found that inosine treatment resulted in less I/R‐induced cardiac cell death, as evidenced by an increased TUNEL positivity in PBS‐treated I/R mice when compared to the sham group (*p* < 0.001) as well as by the reversed TUNEL positivity in inosine‐treated I/R mice (*p* < 0.001) (Figure 6A). To understand how inosine protected against cardiac cell death in mouse hearts after I/R, we used an in vitro model of C2C12 myoblasts that were subjected to oxygen‐glucose deprivation/reoxygenation (OGDR) to simulate an in vivo I/R injury (Figure 6B) and found a dose‐dependent antiapoptotic effect by inosine (Figure 6C). To elucidate how inosine conferred a pro‐survival effect on C2C12 myoblasts against OGDR, we employed two pharmacological inhibitors targeting different downstream pathways of inosine [44]. We found that the inosine decreased cell death and improved cell survival of C2C12 myoblasts subjected to OGDR (*p* < 0.001 and *p* < 0.001, respectively), which was unaffected by the addition of ZM241385 (*p* = 0.4126 and *p* = 0.4507, respectively) (Supporting Information S1: Figure S6), the aforementioned A2ARi that blocks the inosine‐A2AR pathway. By comparison, the antiapoptotic effect of inosine was blocked by forodesine (*p* < 0.001 and *p* < 0.01, respectively) (Figure 6D and Supporting Information S1: Figure S6), an inhibitor of purine nucleoside phosphorylase (PNP) that converts inosine to hypoxanthine and ribose‐1‐phosphate (R1P) within the cells [45, 46], suggesting that the antiapoptotic effect of inosine is mediated via inosine catabolism.

Previous studies have reported that upon the breakdown of inosine, hypoxanthine, and R1P can be utilized in the purine salvage pathway to recover the ATP reservoir. Accordingly, to define the potential catabolic fate in ischemic hearts, we next employed siRNA‐mediated knockdown of key enzymes for each process and evaluated whether the interruption of a particular process would significantly affect the pro‐survival effect of inosine in C2C12 myoblasts. The results showed that the inhibition of OGDR‐induced cell death by inosine was unaffected by siRNA‐mediated knockdown of phosphoglucomutase 2 (*Pgm2*) (*p* = 0.9981) or phosphoribosyl pyrophosphate synthetase 1 (*Prps1*) (*p* = 0.7907). By contrast, siRNA‐mediated knockdown of either hypoxanthine‐guanine phosphoribosyltransferase (*Hprt1*) (*p* < 0.001) or adenine phosphoribosyltransferase (*Aprt*) (*p* < 0.001) that are central enzymes for purine salvage pathway led to the loss of inosine′s protection against OGDR‐induced cell death [47] (Figure 6E and Supporting Information S1: Figure S7A–D), suggesting that inosine exerts an antiapoptotic effect via the conversion to intermediate metabolites feeding into purine salvage pathway for ATP generation. Consistently, we found that the addition of inosine was followed by an increase in the adenosine monophosphate (AMP) levels of inosine in C2C12 myoblasts subjected to OGDR (*p* < 0.001) (Figure 6F). Likewise, increased ATP levels were detected upon the addition of inosine in C2C12 myoblasts subjected to OGDR (*p* < 0.001), whereas this effect was inverted upon PNP inhibition on inosine catabolism by the addition of forodesine (*p* < 0.001) (Figure 6G). These data suggest that inosine conferred antiapoptotic effects in C2C12 myoblasts subjected to ODGR by serving as an alternative source for ATP generation through the purine salvage pathway.

Finaly, we investigated whether inosine may serve as an alternative fuel source during I/R by measuring the levels of ATP in the infarcted heart tissues of different groups. We found that ATP levels were significantly lower in mouse hearts after ischemia and within 12 h after reperfusion as compared to that of the Sham group (Figure 6H). Subsequently, we detected significantly higher ATP levels in I/R mouse hearts treated with inosine as compared to those treated with PBS at both 30 min and 24 h after reperfusion (*p* < 0.001 and *p* < 0.01, respectively) (Figure 6I,J), thus proving that inosine improved the energy metabolism by preserving ATP levels in the heart after I/R. Taken together, these data suggest that inosine attenuates cardiac cell death by serving as an alternative fuel source in mouse hearts upon I/R.

## DISCUSSION

This study revealed the potential of a commonly utilized probiotic, *B. infantis*, known for its use in food fermentation and infant probiotic products [48], in reducing cardiac injury after I/R. Notably, *B. infantis* significantly improved cardiac function and mitigated heart injury resulting from I/R, including reduced cell death and inflammation, demonstrating its pleiotropic benefits. Concurrently, we observed a substantial increase in inosine levels in the circulatory system after the gavage with *B. infantis* and discovered that inosine recapitulated the cardioprotective effects of the bacterial strain against I/R. Our transcriptomic investigation revealed that inosine mitigated I/R‐induced inflammation and reduced cardiac cell death. Further mechanistic studies revealed that inosine exerted immunomodulatory effects on immune cells, such as including DCs and NK cells, through the classical adenosine A (2A) receptor. Additionally, inosine displayed antiapoptotic properties by enhancing ATP levels via the purine salvage pathway. Collectively, our findings present a novel therapeutic approach involving prophylactic administration of *B. infantis* or its metabolite inosine for treating myocardial I/R injury.

Cardiac metabolism holds significant implications for the treatment of I/R after MI, as it undergoes profound alterations during this process [49, 50]. Specifically, the heart cells face a drastic depletion in energy substrates, such as fatty acids, and accumulate harmful levels of metabolic intermediates and reactive oxidative species (ROS), potentially leading to further cellular damage and inflammation [51, 52]. To combat these metabolic impairments, the activation of various pathways, including glycolysis, glucose oxidation, ketone oxidation, hexosamine biosynthesis, and deacetylation, has been proposed as a strategy to restore cardiac metabolic balance after I/R [53, 54]. Supplementing key energy substrates, such as ATP, inosine, and adenosine, has been shown to preserve cardiac function by stimulating adenine resynthesis during ischemic conditions [55, 56]. Indeed, our findings reveal that inosine elevated cellular ATP levels in stressed myocardial cells by its intracellular flux into the purine salvage pathway, ultimately leading to ATP production. In addition, inosine has been reported to activate energy consumption in other tissues, such as brown adipocytes, thereby regulating energy homeostasis and potentially alleviating metabolic disorders like obesity and diabetes, which are significant risk factors for CVDs [57, 58, 59]. Combining these insights with our findings, it is plausible that *B. infantis* and its metabolite inosine may possess both short‐term and long‐term beneficial effects when given to patients with a higher risk of developing MI.

In addition to its antiapoptotic effects on I/R cardiac cells, inosine also exhibits anti‐inflammatory effects via the modulation of the immune cells participating in the I/R‐induced inflammation response. Our research revealed that while inosine treatment did not alter the number or polarization of macrophages post‐I/R, it significantly reduced the levels of pro‐inflammatory cytokines such as IL‐1β and IL‐6. Furthermore, inosine significantly reduced the numbers of other immune cell groups, including DCs and NK cells. Notably, DCs are known to respond to necrosis in postischemic heart tissues, triggering autoimmune responses and inflammation [60, 61]. However, the precise mechanisms underlying how inosine exerts cardioprotection by modulating these diverse immune cell types to achieve an overall therapeutic effect remain to be thoroughly investigated. Moreover, our recent study has demonstrated that prophylactic administration of *Lactobacillus reuteri* or its metabolite GABA mitigates macrophage‐mediated cardiac inflammation, thereby alleviating cardiac dysfunction after I/R [62]. Collectively, these studies shed light on the microbiota‐based therapeutic approaches for treating I/R, where the use of probiotics in conjunction with traditional therapies holds the potential to reduce myocardial injury and improve patient outcomes.

Our study has several limitations. While previous studies have reported that bacteria such as *Bifidobacterium pseudolongum* [35], in addition to *B. infantis* can produce inosine to potentially enhance the antitumor effect of immune checkpoint blockade, further investigation is required to determine if *B. infantis* alone or in combination with other strains is the most efficient and feasible approach for inosine‐based probiotic supplementation. Additionally, as the administration of *B. infantis* or inosine only partially restored cardiac function after I/R, future studies should explore whether combined use of *B. infantis* and inosine could yield an additive cardioprotective effect. Lastly, the potential protective benefits of *B. infantis* or inosine in other CVDs, including chronic heart failure, remain unanswered and merit further research.

## CONCLUSION

In summary, our study demonstrates the prophylactic potential of *B. infantis* and its metabolite inosine in mitigating I/R injury after acute MI. In particular, inosine exerts an anti‐inflammatory effect on immune cells, particularly DCs and NK cells, and functions as an alternative energy source to alleviate cardiac cell death following I/R. This study thus offers crucial insights into the translational application of *B. infantis* and its metabolite inosine in preventing and potentially treating myocardial I/R injury and even a broader range of CVDs. Additionally, it opens the door for the utilization of *B. infantis* and its metabolite inosine in addressing a broader spectrum of inflammatory and metabolic impairments.

## METHODS

### Animals

All the mice used in this study were obtained from SPF Biotechnology. C57BL/6J male mice aged 8–10 weeks were caged and fed an autoclaved diet under a 12‐h light cycle in SPF animal facilities and all the animal experiments were approved by the Institutional Care and Ethical Committee of the Institute of Zoology, Chinese Academy of Sciences.

### Bacterial strains and oral administration

To deplete the gastrointestinal microbiota of mice, vancomycin (0.125 mg/mL/day) (1404‐94‐9, Sigma‐Aldrich), neomycin (0.25 mg/mL/day) (1405‐10‐3, Macklin), metronidazole (0.25 mg/mL/day) (443‐48‐1, Solarbio), and ampicillin (0.25 mg/mL/day) (7177‐48‐2, Solarbio) were mixed in drinking water and given to mice ad libitum for 7 days [63]. To enhance the taste of the mixture, aspartame (0.1 mg/mL/day) (A801109, Macklin) was added (A801109, Macklin). In the following experiments, mice were kept in a completely bacteria‐free environment after being treated with an antibiotic cocktail.

*B. infantis* strains were purchased from the China Center of Industrial Culture Collection (CICC 6069) and maintained at −80°C in 20% glycerol before use. Before the experiments, the bacteria were propagated twice in *Bifidobacterium* MRS broth (Hopebio) overnight at 37°C. Cell pellets were resuspended in PBS after centrifugation at 8000*g* for 10 min at 4°C to obtain a density of 1 × 10^8^ CFU per mL. For the duration of the study, mice were gavaged daily with 200 μL suspension solution.

### Quantification of 16S rDNA

A total of 16S rDNA extracted from the fecal samples was performed according to the manufacturer's instructions with a DNeasy PowerSoil Pro Kit (47016, QIAGEN, Germany) to assess antibiotic depletion. The bacterial 16S rRNA gene was then amplified (Fw 5′‐TCCTACGGGAGGCAGCAGT‐3′; Re 5′‐GGACTACCAGGGTATCTAATCCTGTT‐3′) and *Bifidobacterium infantis* was quantified with species‐specific primers (Fw 5′‐ATACAGCAGAACCTTGGCCT‐3′; Re 5′‐GCGATCACATGGACGAGAAC‐3′). Quantitative real‐time PCR was conducted on a 7500 Fast Real‐Time PCR System using the KAPA SYBR FAST Kit (KK4601, KAPA). Real‐time PCR was used to determine the amount of amplified DNA using a 16S rDNA standard curve derived from serial dilutions of *Escherichia coli* DNA.

### I/R studies in vivo and echocardiography (Echo)

Anesthetized with 2% isoflurane, the mice′s chest cavities were opened at the fourth intercostal space and the pericardium was removed. Afterward, the left anterior descending (LAD) coronary artery was then ligated for 40 min using silk ligature and then released to let the ischemia area repercussed.

To evaluate mouse cardiac function after I/R, a Visual Sonics Vevo 3100 imaging system (Visual Sonics, Inc.) was used to perform transthoracic echocardiography with a 30‐MHz transducer (MX400). Two‐dimensional targeted M‐mode traces were obtained at the level of the papillary muscle in all mice anesthetized with 1.5% isoflurane in 100% oxygen. LVEF, LVFS, LVEDD, and LVESD were measured using the cardiac echocardiography software and calculated by ImageJ (ImageJ2, Fiji) from three separate cardiac cycles.

### Histological analysis of mouse heart

After being fixed in 4% paraformaldehyde (PFA) (DF0131, Leagene Biotechnology), the heart tissue was dehydrated using graded alcohols, embedded in paraffin wax and sectioned at a thickness of 5 µm. After being deparaffinized in xylene, the sections were rehydrated in ethanol.

According to the instructions provided by the manufacturer, TUNEL staining (A113‐03, TUNEL Apoptosis Detection Kit, Vazyme) was used to analyze apoptosis. A picrosirius red staining and a Masson trichrome staining were used to assess cardiac fibrosis. For picrosirius red staining, sections were rehydrated and incubated in picrosirius red dye (DC0041, Leagene Biotechnology) for 60 min at room temperature, followed by 10 min with Weigert iron hematoxylin (ZLI‐9610, ZSGB‐BIO). After washing in running water, the sections were dehydrated through ethanol serial and xylene and mounted with neutral balsam (N116470, Aladdin). For Masson trichrome′s staining, sections were rehydrated and immersed in a Weigert iron hematoxylin (ZLI‐9610, ZSGB‐BIO) solution for 10 min and followed by Biebrich Scarlet‐Acid Fuchsin (TRM‐1, Trichrome Stain Kit, Scy Tek) for 15 min at room temperature. Afterward, the sections were incubated at room temperature in a phosphotungstic‐phosphomolybdic acid solution (1:1) (TRM‐1, Trichrome Stain Kit, Scy Tek) for 5 min. After immersion in aniline blue solution (TRM‐1, Trichrome Stain Kit, Scy Tek) for 10 min at room temperature and 1% acetic acid (TRM‐1, Trichrome Stain Kit, Scy Tek) for 1–2 min, the sections were dehydrated in an ethanol series and xylene and mounted using a neutral balsam mounting medium (N116470, Aladdin). With an Axio Observer Z1 widefield microscope, images of stained sections were acquired. Using ImageJ (ImageJ2, Fiji), blue‐colored areas indicate areas of cardiac fibrosis.

For immunohistological staining, rehydrated sections were incubated with 3% H_2_O_2_ at room temperature for 10 min, followed by sodium citrate buffer treatment to remove antigens. The sections were treated with blocking buffer (5% bovine serum, A8020, Solarbio) for 1 h at room temperature, then incubated with primary antibodies against IL‐1β (1:200, 16806‐1‐AP, Proteintech) overnight at 4°C. The next day, after washing three times in PBS for 10 min each, the sections were incubated with biotinylated goat anti‐rabbit secondary antibody (SP‐0022 SP Kit, Biossa) for 1 h and followed by streptavidin horseradish peroxidase (HRP) (SP‐0022 SP Kit, Bioss) for 30 min at room temperature. The sections were visualized using 3,3′‐diaminobenzidine tetrahydrochloride (DAB) (8059, Cell Signaling Technology) and counterstained with hematoxylin before being dehydrated and mounted. A negative control was prepared using IgG as the primary antibody. Using ImageJ (ImageJ2, Fiji), we quantified IL‐1β signals from stained sections acquired with an Olympus microscope IX73.

### Measurement of inosine concentration by HPLC‐MS

Inosine concentration was measured by using high‐performance liquid chromatography‐mass spectrometry (HPLC‐MS) as previously described [64]. The level of Inosine in heart tissue was measured by using HPLC‐MS. Heart tissues were extracted in methanol and analyzed using an Agilent 1290 Infinity II LC system coupled to an Agilent 6495 triple quadrupole LC/MS (Agilent 1290‐6495, Agilent Technologies). The samples were separated using a 3.5‐μm ZORBAX Eclipse Plus C18 (100 mm × 2.1 mm). The mobile phase, consisting of 100% acetonitrile (solvent A) and 100% water (solvent B), was linearly eluted from 30% solvent A and 70% solvent B (*t* = 0 min) to 100% solvent A (*t* = 70 min). The flow rate of the mobile phase was 0.8 mL/min, and the temperature of the column oven was 30°C.

### RNA sequencing

Sequencing libraries were generated using Illumina® Stranded mRNA Prep, Ligation from Illumina, following the manufacturer's instructions. Sequencing of the qualified libraries was conducted on Illumina platforms using the PE150 strategy (Majorbio), according to the effective library concentration and the amount of data that was required. R package DESeq.2 (version 1.12.3) was used to analyze differentially expressed genes (DEGs), and the fold change was multiplied by 2 to identify DEGs, and an adjusted *p* value of 0.05 was used to identify them (Benjamini‐Hochberg method) [65, 66]. With variance stabilizing transformation of the read counts, principal component analysis (PCA) was performed using the prcomp function of the R Stats Package (version 3.6.2). DEGs were subjected to volcano plots, heatmaps, pathway and gene enrichment analyses. The volcano plots and the histograms were visualized with the R package ggplot2 (version 3.4.2). Heatmaps were visualized with the R package pheatmap (version 1.0.12). GO enrichment was determined with clusterProfiler (version 3.18.1) using R. The results of GO enrichment were visualized with ComplexHeatmap (version 2.10.0). GSEA was performed using the clusterProfiler (version 3.18.1). The results of GSEA were visualized with R package GseaVis (version 0.0.9) [67, 68].

### Measurement of cytokine levels by ELISA

For measurement in the serum, blood was withdrawn from the mouse facial vein at indicated time points in the study, left undisturbed at 4°C for 4 h to clot, and centrifuged at 1000*g* for 10 min. Serum from the resultant supernatant was collected for downstream analyses. The levels of serum cTnT (F7649B, Yutong), IL‐1β (RK00006, Abclonal, China), and IL‐6 (RK00008, Abclonal) were detected by using ELISA kits following the manufacturer's instructions.

### Flow cytometry analysis of immune cells in the heart

As described previously [69], single‐cell suspensions of mouse hearts were prepared. After injecting EDTA buffer (130 mM NaCl, 5 mM KCl, 0.5 mM NaH_2_PO_4_, 10 mM HEPES, 10 mM Glucose, 10 mM 2,3‐Butanedione monoxime, 10 mM Taurine, 5 mM EDTA) into the mouse heart, the inferior vena cava was opened in the cavity. After clamping the aorta, the heart was isolated and perfused with EDTA buffer, Perfusion Buffer (130 mM NaCl, 5 mM KCl, 0.5 mM NaH_2_PO_4_, 10 mM HEPES, 10 mM Glucose, 10 mM 2,3‐Butanedione monoxime, 10 mM Taurine, 1 mM MgCl_2_), and collagenase buffer (0.5 mg/mL Collagenase 2, LS004176, Worthington; 0.5 mg/mL Collagenase 4, LS004188, Worthington) in sequence. Heart tissues were pulled gently into 1 mm^3^ piece using forceps and dissociated by gentle pipetting in a dish with collagenase buffer after the clamp was removed. By adding stop buffer, enzymes were deactivated after 1 h of digestion (perfusion buffer with the addition of sterile FBS). Cell suspensions were filtered through 100 μm strainers, and cardiomyocytes were gravity‐settled after being filtered for 20 min. The supernatant containing other types of cells, including immune cells, was collected and centrifuged at 300*g* for 5 min at 4°C. As described previously [70], Cell pellets were resuspended in 80 μL PBS for the following analysis. Cells were stained with monoclonal antibodies for 30 min after being treated with Fcg‐blocking antibody anti‐mouse CD16/32 for 10 min at 4°C. All immune cells were gated by CD45^+^. Neutrophils were gated as CD11b^+^ Ly6G^high^. CD11b^+^ macrophages were gated as CD11b^+^F4/80^+^. DCs and NK cells were gated by CD11c^+^I‐Ab^+^Ly6C^−^Ly6G^− ^and NK1.1^+^CD3^−^, respectively. All samples were analyzed on a FACS Canto II Cell analyzer (BD Biosciences). Analysis of acquired data was performed using the FlowJo software (FlowJo 10.8.1).

The antibodies used were as follows: Fcg‐blocking antibody anti‐mouse CD16/32 (101319, BioLegend), BV421 anti‐mouse CD45 (103134, BioLegend), BV510 anti‐mouse CD11b (101263, BioLegend), APC anti‐mouse CD3 (100235, BioLegend), BV605 anti‐mouse Ly6C (128036, BioLegend), FITC anti‐mouse Ly6G (127605, BioLegend), PE anti‐mouse F4/80 (123109, BioLegend), PE anti‐mouse NK1.1 (156503, BioLegend), PE anti‐mouse CD11c (117307, BioLegend), and APC anti‐mouse I‐Ab (116417, BioLegend).

### OGDR induction

C2C12 mouse myoblasts were grown in proliferation medium (PM), which is Dulbecco's modified Eagle's medium (DMEM, BI) with 10% fetal bovine serum and 1% antibiotic/antimycotic. Cells were plated at a density of 2 × 10^5^ cells/mL in PM during proliferation. For the normoxia group, cells were kept in the normal incubator with PM for 10 h. For the OGDR group, cells were incubated in AVATAR incubators (XCell Biosciences) with 0.1% O_2_, 5% CO_2_, 94.9% N_2_, and DMEM without FBS or glucose for 8 h and taken out for another 2 h under normoxic conditions.

### Cell viability assay

Assays were conducted using either 96‐well or 12‐well plates, in which the cells were removed from the culture medium or rinsing solution before testing. To each well of a 96‐well plate, 100 μL of Alamar Blue solution (10% in PBS, A7631, Solarbio) was added, and the plates were incubated at 37°C for 2 h. Using a Cytation 5 fluorescence multi‐well plate reader (Agilent Technologies) with the excitation/emission wavelengths at 545/590 nm, the fluorescence was measured in a new plate after incubation. For the Live/Dead assay, 100 μL (for 96‐well plates) of Calcein‐AM/Propidium Iodide (PI; 1 M Calcein‐AM, 1 μg/mL PI) solution was added to each well, and the plates were incubated at 37°C for 0.5 h. Fluorescence intensities were measured in a new plate using a Cytation 5 fluorescence multi‐well plate reader with the excitation/emission wavelengths set at 494/517 nm for Calcein and 535/617 nm for PI. Andor DragonFly 600 was used to capture the images.

### siRNA‐mediated gene knockdown

siRNAs were purchased from JTSBIO Co. siRNA‐mediated knockdown of target genes was performed in C2C12 with ON‐TARGET plus control siRNAs with the following sequences:

siNC: 5′‐GCUUGUUGCAAAUAUGCUAUC‐3′;

siRNA‐*Pgm2*: 5′‐CGAGUUUCCAACUGUCAAAUA‐3′;

siRNA‐*Prps1*: 5′‐GCUUGUUGCAAAUAUGCUAUC‐3′;

siRNA‐*Hprt1*: 5′‐CGUUUGUGUCAUUAGUGAAAC‐3′;

siRNA‐*Aprt*: 5′‐GCGGCAAGAUCGACUACAUCG‐3′.

Transfection of siRNAs in C2C12 myoblasts was performed using JetPrime transfection reagent (101000046, Polyplus) according to the manufacturer's instructions.

### RNA extraction and quantitative RT‐PCR

TRIzol Regent (15596018, Thermo Fisher Scientific) was used to extract total RNA from cells or heart tissues from mice. RevertAid Master Mix (M1631, Thermo Fisher Scientific) was used for complementary cDNA synthesis. TB Green Premix Ex Taq™ (Tli RNaseH Plus) (RR420D, Takara) was used to access PCR amplification products. The mRNA levels of genes were normalized to those of *18S* or *Gapdh*. The sequences of primers used for quantitative RT‐PCR are listed below.

*Pgm2* Fw: 5′‐AGTGAAGACGCAGGCATATCC‐3′;

*Pgm2* Re: 5′‐GGCTCCACGGTAGAGACGA‐3′;

*Prps1* Fw: 5′‐ACTTATCCCAGAAAATCGCTGAC‐3′;

*Prps1* Re: 5′‐CCACACCCACTTTGAACAATGTA‐3′;

*Hprt1* Fw: 5′‐CAAACTTTGCTTTCCCTGGT‐3′;

*Hprt1* Re: 5′‐TCTGGCCTGTATCCAACACTTC*‐*3′;

*Aprt* Fw: 5′‐CCCTCTTGAAAGACCCGGAC‐3′;

*Aprt* Re: 5′‐CTGCGATGTAGTCGATCTTGC*‐*3′;

*Tnf* Fw: 5′‐CACAGAAAGCATGATCCGCGACGT‐3′;

*Tnf* Re: 5′‐CGGCAGAGAGGAGGTTGACTTTCT‐3′;

*18S* Fw: 5′‐GCTTAATTTGACTCAACACGGGA‐3′;

*18S* Re: 5′‐AGCTATCAATCTGTCAATCCTGTC‐3′;

*Gapdh* Fw: 5′‐CATCACTGCCACCCAGAAGACTG‐3′;

*Gapdh* Re: 5′‐ATGCCAGTGAGCTTCCCGTTCAG‐3′.

### Measurement of AMP levels by HPLC

The AMP content of C2C12 cells was determined using the corresponding content test kit (Solarbio, BC1024), according to the manufacturer's specifications. The HPLC analysis was performed using the Agilent 1260 HPLC System (Agilent Technologies) with an ultraviolet detector. A Diamonsil Plus C18 column (5 µm, 250 mm × 4.6 mm) was used as the chromatographic column.

### Determination of ATP levels

For sample preparation, 200 μL of lysis buffer was added to each well of cells or to 40 mg of heart tissues, followed by homogenization. The lysates were then centrifuged at 12,000*g* at 4°C for 5 min. The resultant supernatants were collected for subsequent measurement of ATP levels in the cells or heart tissues by using a commercially available kit (S0026, Beyotime) following the manufacturer's instructions. ATP luminescence intensities were recorded using BioTek Cytation 7 cell imaging multimode reader (Agilent Technologies), and ATP levels were calculated as nmol/mg protein measured with a BCA protein assay kit (P0012, Beyotime).

### Statistical analysis

All statistical analyses were conducted using GraphPad Prism 8.4 software (Prism Inc.). The two groups were compared using a two‐tailed unpaired Student's *t* test as appropriate. When comparing more than two groups, one‐way or two‐way analysis of variance (ANOVA) was used, followed by the post hoc Tukey test or Dunnett's test. Individual data points or mean ± SEMs are presented. Differences are considered statistically significant at *p* < 0.05.

## AUTHOR CONTRIBUTIONS

Hao Zhang, Jiawan Wang, Jianghua Shen, Hailong Yuan, Xuan Zhang, Xu Liu, Ying Yu, Xinran Li, Zeyu Gao, and Yaohui Wang did the experiments and analyzed the data. Hao Zhang, Jiawan Wang, and Jianghua Shen drafted the manuscript. Moshi Song and Jun Wang supervised this project and revised the manuscript. All authors have read the final manuscript and approved it for publication.

## CONFLICT OF INTEREST STATEMENT

The authors declare no conflict of interest.

## ETHICS STATEMENT

The ethics application (No. IOZ20190057) was approved by the Institutional Care and Ethical Committee of the Institute of Zoology, Chinese Academy of Sciences.

## Supporting information

 


**Figure S1:** The gavage of *B. infantis* mitigated cardiac injury in mice after I/R.
**Figure S2:** Measurement of serum inosine levels in mice with or without antibiotics treatment.
**Figure S3:** Transcriptional analysis of fatty acid metabolism pathway, immune response pathway, and apoptosis pathway in mouse hearts.
**Figure S4:** Measurement of *Tnf* mRNA levels in the heart tissues after I/R.
**Figure S5:** Inosine did not affect the numbers of neutrophils or CD11b^+^ macrophages one day after I/R.
**Figure S6:** Improvement of cell survival by inosine under oxygen‐glucose deprivation/re‐oxygenation (OGDR) condition was blocked by forodesine.
**Figure S7:** siRNA‐mediated knockdown of genes related to the purine salvage pathway.

## Data Availability

All the sequencing data have been deposited in GSA under submission number CRA016606 (Illumina RNA sequencing), BioProject accession number PRJCA026354 (https://ngdc.cncb.ac.cn/bioproject/browse/PRJCA026354). The data and scripts used are saved on GitHub (https://github.com/xpf10/inosine). Supporting Information (figures, tables, scripts, graphical abstract, slides, videos, Chinese translated version, and updated materials) may be found in the online DOI or iMeta Science http://www.imeta.science/.
