# Correction to “Prophylactic supplementation with *Bifidobacterium infantis* or its metabolite inosine attenuates cardiac ischemia/reperfusion injury”

**DOI:** 10.1002/imt2.70162

**Published:** 2026-07-30

Zhang, Hao, Jiawan Wang, Jianghua Shen, Siqi Chen, Hailong Yuan, Xuan Zhang, Xu Liu, et al. 2024. “Prophylactic Supplementation With *Bifidobacterium infantis* or Its Metabolite Inosine Attenuates Cardiac Ischemia/Reperfusion Injury.” *iMeta* 3, e220. https://doi.org/10.1002/imt2.220

In the originally published Supplementary Information, the source data for 5F contains incorrect entries owing to an unintentional data transfer error. Supplementary Table has now been corrected. We apologize for this oversight.

