# Supplementary material for: Prophylactic supplementation with Bifidobacterium infantis or its metabolite inosine attenuates cardiac ischemia/reperfusion injury
Source: Imeta. 2024 Jul 2;3(4):e220. doi: 10.1002/imt2.220 (PMC11316933; doi:10.1002/imt2.220)
Supplement: Supplementary file 2 — Figure S1: The gavage of B. infantis mitigated cardiac injury in mice after I/R. Figure S2: Measurement of serum inosine levels in mice with or without antibiotics treatment. Figure S3: Transcriptional analysis of fatty acid metabolism pathway, immune response pathway, and apoptosis pathway in mouse hearts. Figure S4: Measurement of Tnf mRNA levels in the heart tissues after I/R. Figure S5: Inosine did not affect the numbers of neutrophils or CD11b+ macrophages one day after I/R. Figure S6: Improvement of cell survival by inosine under oxygen‐glucose deprivation/re‐oxygenation (OGDR) condition was blocked by forodesine. Figure S7: siRNA‐mediated knockdown of genes related to the purine salvage pathway. [file IMT2-3-e220-s001.docx]

**Supporting information to:**

**Prophylactic supplementation with *Bifidobacterium infantis*** **or its metabolite inosine attenuates cardiac injury after ischemia/reperfusion**

**Running title:** *Bifidobacterium infantis* attenuates myocardial ischemia/reperfusion injury

Hao Zhang^1,2*^, Jiawan Wang^1,3*^, Jianghua Shen^1,2,4*^, Siqi Chen^1,2,4^, Hailong Yuan^1,4,5^, Xuan Zhang^2,6^, Xu Liu^1,2,4^, Ying Yu^2,6^, Xinran Li^1,2,4^, Zeyu Gao^1,4,7^, Yaohui Wang^5^, Jun Wang^2,6#^, Moshi Song^1,2,4,7#^

^1^Key Laboratory of Organ Regeneration and Reconstruction, State Key Laboratory of Membrane Biology, Institute of Zoology, Chinese Academy of Sciences, Beijing 100101, China

^2^University of Chinese Academy of Sciences, Beijing 100049, China

^3^Beijing Chao-Yang Hospital, Department of Anesthesiology, Beijing 100020, China

^4^Beijing Institute for Stem Cell and Regenerative Medicine, Beijing 100101, China

^5^Joint National Laboratory for Antibody Drug Engineering, Henan University, Kaifeng 475004, China

^6^CAS Key Laboratory of Pathogenic Microbiology and Immunology, Chinese Academy of Sciences, Beijing 100101, China

^7^Institute for Stem Cell and Regeneration, Chinese Academy of Sciences, Beijing 100101, China

*These authors contributed equally: Hao Zhang, Jiawan Wang, and Jianghua Shen

#Correspondence: [junwang@im.ac.cn](mailto:junwang@im.ac.cn) (Jun Wang) and [songmoshi@ioz.ac.cn](mailto:songmoshi@ioz.ac.cn) (Moshi Song).

**Supplementary figures**

**Figure S1** **The gavage of *B. infantis* mitigated cardiac injury in mice after I/R.** (A) Representative echocardiographic images of mouse hearts at baseline and two weeks after I/R. (B) Quantitative data of left ventricular ejection fraction (LVEF), left ventricular fractional shortening (LVFS), left ventricular end-diastolic dimension (LVEDD), and left ventricular end-systolic dimension (LVESD). *n =* 11 each group. Data are shown as the mean ± SEMs. ***p <* 0.01; ****p <* 0.001 (one-way ANOVA with post hoc Tukey test).

**Figure S2 Measurement of serum inosine levels in mice with or without antibiotics treatment.** HPLC measurement of serum inosine levels in mice treated with PBS or antibiotics (ABX). *n =* 5 each group. *ns*, not significant (Student’s *t*-test).

**Figure S3 Transcriptional analysis of fatty acid catabolism pathway, immune response pathway and apoptosis pathway in mouse hearts.** (A) Gene set enrichment analysis (GSEA) plots showing the enrichment of gene sets associated with fatty acid metabolism, positive regulation of cytokine production and apoptosis in PBS *+* I/R group when compared to Sham group. The normalized enrichment scores (NES), *p*-value, and adjusted *p*-value are shown on the plots. (B-D) Histograms showing the changes in the expression levels of genes related to fatty acid beta oxidation (B), innate immune response activating signaling pathway (C), and apoptosis (D) in PBS *+* I/R vs. Sham groups and in Inosine *+* I/R vs. PBS *+* I/R groups. The blue columns denote downregulated gene expression, the red columns denote upregulated gene expression, and the grey columns denote gene expression without significant difference. |Fold change| > 2 and adjusted *p*-value < 0.05.

**Figure S4 Measurement of *Tnf* mRNA levels in the heart tissues after I/R.** *Tnf* mRNA levels in the heart tissues of mice in Sham, PBS *+* I/R, and Inosine *+* I/R groups. *n =* 4 each group. Data are shown as the mean ± SEMs. ***p <* 0.01; ****p <* 0.001 (one-way ANOVA with post hoc Tukey test).

**Figure S5 Inosine did not affect the numbers of neutrophils or CD11b^+^ macrophages one day after I/R.** (A) Gating strategy identifying cardiac immune cells, including neutrophils and CD11b^+^ macrophages. (B) Representative dot plots of CD45^+^ immune cells, neutrophils and CD11b^+^ macrophages in heart tissue one day after I/R (left panel). Quantification of the percentages of CD45^+^ cells, neutrophils and CD11b^+^ macrophages in all non-myocytes (right panel). Sham group, *n =* 4; PBS *+* I/R group, *n =* 5; Inosine *+* I/R group, *n =* 3. Data are shown as the mean ± SEMs. ***p <* 0.01; *ns*, not significant. (one-way ANOVA with post hoc Dunnett’s test). (C) Representative dot plots of neutrophils, CD11b^+^ macrophages, DCs and NK cells in heart tissues seven days after I/R. Ellipses or rectangles indicate the corresponding types of cells. DCs, dendritic cells; NK cells, natural killer cells.

**Figure S6** **Improvement of cell survival by inosine under** **oxygen glucose deprivation/re-oxygenation (OGDR) condition was blocked by forodesine.** Cell viability detected by Alamar Blue of C2C12 myoblasts treated with ZM241385 or forodesine in the presence or absence of 1 mM inosine under normoxic and OGDR conditions. *n* ≥ 4 for each group. Data are shown as the mean ± SEMs. ****p <* 0.001; *ns*, not significant (two-way ANOVA with post hoc Tukey’s test).

**Figure S7 siRNA-mediated knockdown of genes related to the purine salvage pathway.** (A-D) Quantitative PCR showing gene silencing efficiency of siRNAs targeting *Pgm2* (A), *Prps1* (B), *Hprt1* (C), and *Aprt* (D) in C2C12 myoblasts. A negative control siRNA (siNC) was used as a control. The mRNA expression levels were normalized to 18S rRNA. *n =* 3 biological replicates for each group. Data are presented as the mean ± SEMs. **p <* 0.05; ***p <* 0.01; ****p <* 0.001 (Student’s *t*-test).
